# A Blooming-Region-Aware Directional Weighting Filter for Correcting Blooming-Induced Background Depth Distortion in AMCW-ToF LiDAR Imaging of a High-Reflectance Object

**DOI:** 10.3390/s26154997

**Published:** 2026-08-06

**Authors:** Ju-Han Yoon, Woo-Jung Lim, Wooram Kim, Won Chegal, Sung-Hyun Lee

**Affiliations:** 1 Department of Electronic Engineering, Hanbat National University, Daejeon 34158, Republic of Korea; 20211073@edu.hanbat.ac.kr (J.-H.Y.); 20231235@edu.hanbat.ac.kr (W.-J.L.); 2 Semiconductor and Display Metrology Group, Korea Research Institute of Standards and Science (KRISS), Daejeon 34113, Republic of Korea; kwr0704@kriss.re.kr (W.K.); wchegal@kriss.re.kr (W.C.)

**Keywords:** high-reflectance target, blooming artifact, time-of-flight (ToF), light detection and ranging (LiDAR), amplitude-modulated continuous-wave (AMCW), blooming-induced depth error correction, directional weighting filter

## Abstract

When measuring a high-reflectance target using time-of-flight LiDAR (ToF LiDAR), strong reflected optical signals are generated. Pixels receiving such strong reflected optical signals become saturated, and intensity and depth distortions occur even around the saturated pixels owing to crosstalk, internal lens scattering, and related effects. Previous studies on depth distortion in ToF cameras and LiDAR caused by such blooming have mainly focused on removing distorted background pixels, which limits their ability to directly correct background depth distortion around high-reflectance targets. In this paper, we propose a blooming-region-aware directional weighting filter method that corrects blooming-induced background depth distortion around a high-reflectance target using only the intensity map and depth map acquired from a single amplitude-modulated continuous-wave time-of-flight (AMCW-ToF) LiDAR. The proposed method detects the blooming-affected background region based on the intensity map and the gradient information of the intensity map. Subsequently, a window kernel is applied to each correction target pixel within the detected region, and the distorted depth of the correction target pixel is corrected through multi-weight depth filtering that considers the directional relation among pixels inside the kernel. In the validation process, the correction performance was evaluated in terms of mean squared error (MSE) and root mean squared error (RMSE) according to changes in the filtering method, target distance, target direction, and background SNR. The validation results demonstrate that the proposed method reduced the RMSE by an average of 49.811% compared with the blooming-distorted depth under various experimental conditions, indicating that it can mitigate background depth distortion around high-reflectance targets.

## 1. Introduction

Three-dimensional depth information is used as essential sensing information in various intelligent systems, including autonomous mobile robots, object recognition, obstacle detection, mapping, and localization [1,2,3]. In particular, time-of-flight (ToF) cameras and ToF LiDAR are widely used in robotic and autonomous driving systems that require recognition of the geometric structure of surrounding environments because they can directly provide distance information to target objects. Among them, amplitude-modulated continuous-wave (AMCW) time-of-flight (ToF) sensors play an important role in indoor mobile robots, 3D measurement, and object recognition because they can acquire dense depth maps with a compact and cost-effective structure [1,2,4]. In addition, recent studies on miniaturized ToF sensors, SPAD-based ToF image sensors, and RGB-guided ToF depth enhancement have been reported, indicating that ToF sensing hardware and depth processing pipelines are developing toward practical robotics and mobile applications [5,6,7].

However, ToF and LiDAR sensors can generate various depth distortions depending on the measurement environment, scene geometry, surface reflectance, and optical properties. Representative depth artifacts include multipath interference (MPI), phase wrapping, transparent obstacle-induced error, and flying pixels [1,8,9,10,11]. To reduce these artifacts, various studies have been conducted on MPI suppression, flying pixel correction, depth refinement based on raw correlation samples, and model-based or learning-based depth error correction [8,9,10,12,13,14,15]. Optical-scattering compensation has also been investigated in ToF cameras using raw-data calibration models and point-spread-function-based image restoration [16,17]. In conventional imaging, veiling glare caused by multiple scattering within the camera body and lens optics has been characterized using a glare spread function, and deconvolution and direct–indirect separation have been investigated for its removal [18]. These studies address camera-internal scattering or glare using calibrated scattering models, measured spread functions, or controlled optical measurements, whereas the present study focuses on localized blooming-induced background depth distortion using only a single intensity map and depth map. In addition, studies on LiDAR point cloud denoising and reflective object noise removal have been actively conducted [19,20,21,22]. These studies focus on sparse outlier removal, weather-induced noise point removal, dense reflective noise removal, and point cloud segmentation [23,24,25,26,27,28]. However, these previous studies primarily aim to remove point cloud noise or filter noise from the reflective object itself, which differs from studies that define the blooming-distorted surrounding background region as the correction target and restore the depth of this region closer to the actual background depth. Recently, a study that directly addressed LiDAR blooming artifacts caused by high-reflectance objects has also been reported [29]. This study generated blooming artifacts caused by high-reflectance targets using synthetic data and estimated the blooming artifact pixel probability using a deep learning-based segmentation network [29]. However, the main objective of this study was blooming artifact pixel estimation or segmentation, and it did not restore the blooming-distorted surrounding background depth closer to the actual background depth. Therefore, previous studies have insufficiently addressed the definition of blooming-induced background depth distortion as a correction target and the restoration of distorted depth in this region closer to the actual background depth.

Motivated by this problem, this paper defines blooming-induced background depth distortion around high-reflectance targets as a direct correction target. The proposed method uses only the intensity map and depth map acquired from a single AMCW-ToF LiDAR. First, the blooming-affected background region is estimated using a saliency map computed from the intensity map and Sobel-gradient magnitude information, and the correction target region is defined in the depth map based on this estimation. Subsequently, multi-weight depth restoration is performed by jointly considering the pixel label-dependent base weight, Gaussian distance weight, and directional weight for neighboring pixels inside a local kernel, thereby correcting the distorted background depth.

The main contributions of this paper are as follows.

1. This paper defines surrounding background depth distortion caused by high-reflectance blooming as an explicit correction problem. Unlike previous studies that mainly focused on flying pixel removal, object boundary artifact removal, point cloud denoising, or blooming artifact pixel estimation, this study aims to restore the distorted background depth around a high-reflectance object closer to the actual background depth.
2. To address the high-reflectance blooming problem, for which paired training data are insufficient, this paper proposes a blooming-region-aware directional weighting filter framework that uses only the intensity map and depth map acquired from a single AMCW-ToF camera. The proposed method estimates the blooming-affected background region using intensity saliency and gradient information, and performs multi-weight depth restoration by combining the pixel label-dependent base weight, directional weight, and Gaussian distance weight.
3. The correction performance was evaluated quantitatively through comparisons with other depth correction methods and experiments involving variations in target distance, target direction, and background SNR, and qualitatively under target-shape and exposure-time variations. Across the 14 quantitative experimental conditions involving target distance, target direction, and background SNR, the proposed method reduced the RMSE by an average of 49.811% and a maximum of 76.292% relative to the blooming-distorted depth.

The remainder of this paper is organized as follows. Section 2 describes the overall framework of the proposed rule-based blooming correction method and the operating principle of each module. Section 3 describes the experimental setup and evaluation metrics and presents the GT and blooming depth repeatability analysis, directional-weighting ablation, parameter sensitivity analysis, and comparison of depth correction methods. Section 4 evaluates the correction performance under variations in target distance, target direction, background SNR, target shape, and exposure time, and discusses the limitations observed in multiple-object and complex-background scenes. Finally, Section 5 presents the conclusions, limitations, and future research directions.

## 2. Proposed Blooming-Region-Aware Directional Weighting Filter Framework

This section describes the blooming-region-aware directional weighting filter method for correcting blooming-induced background depth distortion that occurs around high-reflectance targets. The proposed method uses the intensity map and depth map acquired from a single AMCW-ToF LiDAR as inputs, and the overall process consists of blooming region detection modules (intensity-based) and a depth correction module (depth-based). First, the main terminology and problem definition are presented, followed by a sequential description of the overall structure of the proposed framework and the detailed operation of each module.

### 2.1. Terminology and Problem Definition

In this study, the following terms are defined to describe the blooming artifact that occurs around high-reflectance targets.

The core region refers to the central saturated region in which valid depth and intensity values are not measured because of strong reflected optical signals from the high-reflectance target [1,29].

The core center is the center point of the core region and is used as the reference coordinate for radial analysis and directional weighting.

The blooming region refers to a region around the core region that has valid depth values but is distorted differently from the actual background depth, and it corresponds to the depth correction target in this study [21,29]. In this paper, the blooming region and the blooming-distorted background region refer to the same region. The proposed method focuses not on restoring the core region itself, but on correcting blooming-induced background depth distortion that occurs around the core region.

The background region is defined as the region excluding the core region and the blooming region. In this study, the background region is regarded as a region less affected by the blooming artifact and is used as a reference region for correcting distorted depth in the depth correction stage. Therefore, neighboring pixels inside a local kernel are classified into blooming neighbors and background neighbors according to their mask labels, and background neighbors are used as depth references with relatively high reliability.

Based on the above definitions, the objective of this study is to estimate the blooming-distorted background region that occurs around high-reflectance targets using an intensity map-based saliency map and gradient information, apply a window kernel to each correction target pixel within the region, and correct the distorted depth through multi-weight depth filtering using information from neighboring pixels inside the kernel. Algorithmic variables used in detail, such as the start point, boundary point, and directional weight, are defined in the description of each module.

### 2.2. Overall Framework

**Figure 1:**
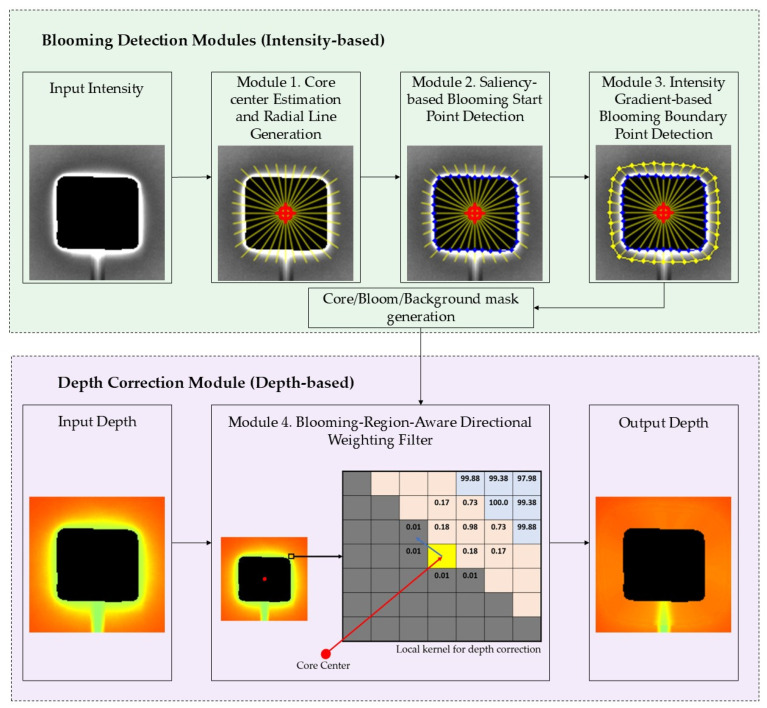
Overall workflow of the proposed blooming-induced background depth distortion correction framework. The framework estimates the core center, radial lines, blooming start points, and boundary points from the intensity map, and then corrects the detected blooming-distorted background region using the blooming-region-aware directional weighting filter.

The overall framework of the proposed method is shown in Figure 1. The blooming detection modules (intensity-based) consist of three modules: core center estimation and radial line generation, saliency-based start point detection, and intensity-gradient-based boundary point detection. First, radial lines are generated based on the core center, and a blooming-distorted background mask is generated by analyzing the saliency map computed from the intensity map and the Sobel-gradient magnitude information along each radial line.

The depth correction module (depth-based) consists of a blooming-region-aware directional weighting filter module. In this stage, the generated blooming-distorted background mask is applied to the corresponding location in the depth map to define the correction target. Subsequently, a window kernel is applied to each correction target pixel within the blooming region, and multi-weight depth correction is performed by jointly considering the pixel label-dependent base weight, directional weight, and Gaussian distance weight of neighboring pixels inside the kernel, thereby correcting the blooming-distorted background depth. This study assumes only the case in which a single high-reflectance target exists in the input image, and the target region itself is excluded from the depth correction target because saturation or invalid measurement occurs in that region. Therefore, the correction target of the proposed method is not the high-reflectance target itself, but the blooming-affected background region around it. The detailed operation of each module is described sequentially in the following sections.

### 2.3. Core Center Estimation and Radial Line Generation

Module 1 in Figure 1, core center estimation and radial line generation, is a preliminary process for analyzing the intensity map along radial lines with respect to the core center in order to estimate the blooming-distorted background region. Blooming artifacts tend to spread from the high-reflectance target toward the surrounding background direction. To analyze the intensity map while accounting for this geometric characteristic of blooming artifacts, the core center is used as the starting point of radial lines for estimating the start and boundary points, and it is also used as the reference coordinate for calculating the directional weight in the subsequent depth correction module. The core center estimation and radial line generation module estimates the core center from the core region using a distance transform and generates radial lines that start from the core center and pass linearly through both the blooming-distorted background region and the undistorted background region.

Before estimating the core center, a core mask was generated using pixels for which valid intensity values were not measured because of saturation in the intensity map. First, pixels with an intensity value of zero were defined as core candidates. For each candidate, a surrounding ring region was generated by applying dilation with a radius of 8 pixels to each candidate region, and the candidate with the highest mean intensity in the corresponding ring region was selected as the final core mask. This process uses the characteristic of blooming in which relatively high intensity values appear around the saturated region of a high-reflectance target, thereby selecting the core region corresponding to the actual high-reflectance target. This process assumes that a single high-reflectance target causing blooming is present within the region of interest (ROI) of the input image. Extending the method to scenes with multiple high-reflectance targets would require each target to be detected and processed separately, which will be investigated in future work.

The core center is estimated using a distance transform inside the core region where saturation or invalid measurement appears. The distance transform is a method that calculates the distance from each pixel inside a region to the nearest boundary, where pixels closer to the boundary have smaller values and pixels closer to the center have larger values [30]. Therefore, the location with the largest distance value can be regarded as a candidate for the core center. In the proposed method, core pixels whose distance transform values are greater than or equal to 99% of the maximum value and differ from the largest distance transform value by 0.5 or less are selected as core center candidate pixels. The position coordinates of these pixels are extracted, and the median of these coordinates is used as the core center. Compared with simply using the pixel with the largest distance transform value as the core center, this approach can estimate the center point more stably even when the shape of the high-reflectance target is not uniform.

After the core center is determined, radial lines with equal angular intervals are generated in the 360° direction using the corresponding coordinate as the origin. In this study, 360° was divided into 32 directions, and a total of 32 radial lines were used. Each radial line is a 1D profile sampled outward from the core center and is used as a reference path for analyzing the saliency map and Sobel gradient map in the subsequent steps. The radial lines pass through the core region, blooming-distorted background region, and undistorted background region, thereby enabling the analysis of blooming-related values appearing in the saliency map and Sobel gradient map obtained from the intensity map along the radial lines.

### 2.4. Saliency-Based Blooming Start Point Detection

**Figure 2:**
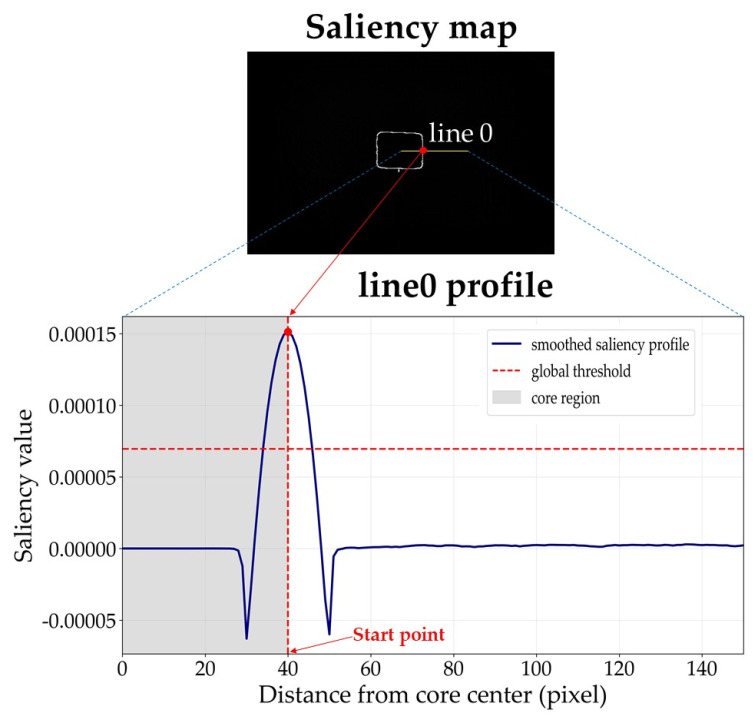
Saliency-based blooming start-point detection along a radial line. The saliency profile is sampled from the reference center along the selected radial line, and the first position where the smoothed saliency profile exceeds the global threshold is defined as the blooming start point.

The purpose of Module 2 in Figure 1, saliency-based blooming start point detection, is to estimate the start point at which the blooming influence begins based on the core center and radial lines defined in Module 1. In Module 2, a saliency map is computed from the intensity map, and the point at which the saliency value first exceeds a threshold along each radial line is detected. The blooming start point estimated in this manner is subsequently used as the blooming-distorted background start point for constructing the blooming-distorted background mask. Figure 2 shows the process of detecting the start point based on the global threshold in the saliency profile of a selected radial line.

In the intensity map, the core region forms pixel values that are clearly distinguished from those of the blooming-distorted background region. In this context, the saliency map obtained from the intensity map emphasizes intensity variations that appear abruptly at specific locations; therefore, it is advantageous for highlighting the start point of the blooming-distorted region that occurs around a high-reflectance target [31,32].

The saliency map is computed using a frequency-domain residual spectrum [32]. First, a 2D Fourier transform is applied to the input intensity map to convert it into the frequency domain, and the result is separated into an amplitude spectrum and a phase spectrum. Subsequently, the log amplitude spectrum of the amplitude spectrum is computed.

In this study, Savitzky–Golay smoothing is applied to the log amplitude spectrum to estimate a smooth frequency trend. The residual spectrum is then computed by removing this trend from the original log amplitude spectrum. The residual spectrum suppresses the global background structure and emphasizes local intensity variations that appear around blooming [32]. Finally, the residual spectrum is combined with the original phase spectrum, and an inverse Fourier transform is performed to generate the saliency map. The saliency map obtained in this manner emphasizes points at which the intensity brightness changes abruptly due to blooming and is used to determine the blooming start point along each radial line.

The point at which the saliency value exceeds the threshold along a radial line is determined as the start point. First, a global saliency threshold is set using an upper percentile value of the saliency map. In this study, the 99.7th percentile value of the saliency map was used as the threshold. In each line, the location at which the saliency value first exceeds the global threshold after exiting the core region is defined as the start point. If no location exceeds the global threshold in the corresponding line, the location with the largest saliency value after the core region is used as the blooming start point. This start point is subsequently used, together with the boundary point estimated in the intensity-gradient-based boundary point detection module, to form the blooming-distorted background mask.

### 2.5. Intensity-Gradient-Based Blooming Boundary Point Detection

**Figure 3:**
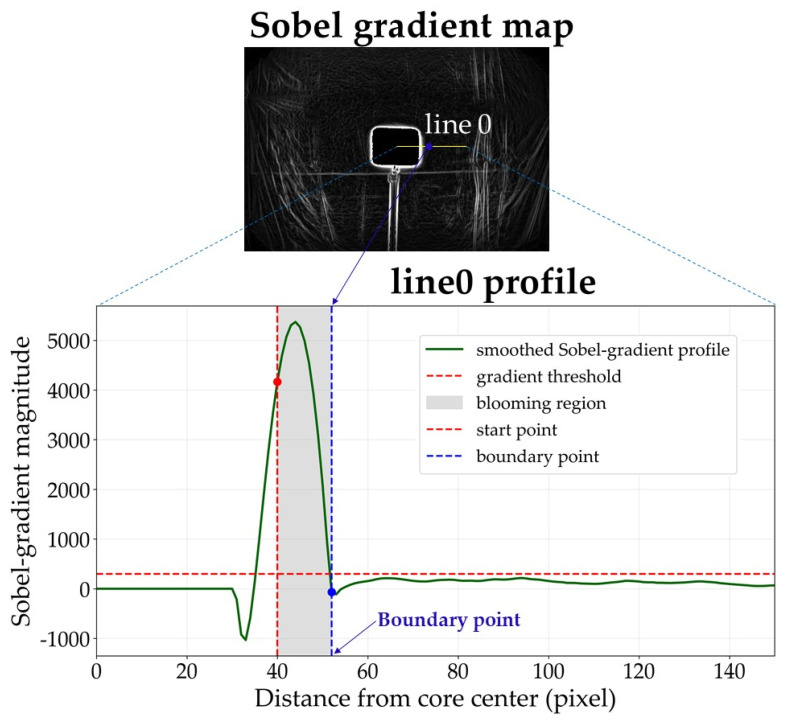
Intensity-gradient-based blooming boundary-point detection along a radial line. The Sobel-gradient profile is sampled from the reference center along the selected radial line, and the first stable low-gradient position after the detected start point is defined as the blooming boundary point.

In Module 3 of Figure 1, intensity gradient-based blooming boundary point detection estimates the boundary point at which the blooming influence becomes sufficiently weak after the start point estimated in Module 2. The saliency map used for the start point emphasizes abrupt intensity variations and is therefore advantageous for determining the blooming start point; however, it is difficult to estimate the outer boundary of the blooming-distorted background region using the saliency map alone. Around a high-reflectance target, after a region with rapidly changing intensity values appears, a region in which the intensity variation becomes gradual appears as the influence of blooming is progressively reduced with increasing distance from the target. This change is more clearly observed in the intensity gradient than in the raw intensity value. Therefore, in this stage, the Sobel-gradient magnitude is computed by applying a Sobel filter to the intensity map, and the boundary point at which the blooming influence is reduced along each radial line is estimated based on this magnitude. Figure 3 shows the intensity-gradient-based boundary point detection process.

To compute the Sobel-gradient magnitude map, Gaussian blur is first applied to the intensity map to reduce local noise. Subsequently, the x-direction and y-direction Sobel gradients are computed, and the two gradient components are combined to generate the gradient magnitude map. This gradient magnitude map emphasizes locations where large intensity changes appear in the image plane and is used as a cue for estimating the outer boundary at which the blooming influence becomes weak.

The boundary point is determined using the gradient profile after the start point detected in Module 2 of Figure 1, saliency-based blooming start point detection. First, in each radial line, the first location at which the gradient magnitude decreases below the threshold and remains below the threshold for a certain length after the start point is defined as the boundary point. In this implementation, the first location at which the Sobel-gradient magnitude remained below 300 for at least 12 consecutive pixels was used as the boundary point. If the location where the gradient simply drops below the threshold once is selected as the boundary point, the result may be sensitive to local fluctuations. Therefore, in this study, the boundary point is defined not as a location where the gradient decreases only once, but as a stable low-gradient location at which the blooming influence is sufficiently weakened. The boundary point computed along each radial line in this manner is regarded as the location where the depth distortion caused by blooming becomes sufficiently weak, and it is used together with the blooming start point computed in Module 2 to construct the final blooming-distorted background mask.

After the blooming start point and boundary point are computed, a two-dimensional blooming mask is generated using the radial line boundary points. The proposed method calculates the radial thickness between the start point and the boundary point computed along each radial line, and uses the median value of the thicknesses obtained from all radial lines as the representative blooming thickness. Subsequently, the start point of each line is retained, whereas the boundary point is adjusted to a location separated from the corresponding start point by the representative thickness to ensure the geometric consistency of the blooming mask. This process is intended to stably construct the shape of the blooming artifact that is continuously formed around the core region while reducing the excessive influence of exceptional blooming boundary points that may occur in some lines on the final mask shape. Although the same median thickness is applied to all radial lines, the independently detected blooming start point on each line is retained. Therefore, positional asymmetry in a biased or tilted blooming region can still be reflected in the final mask through the directional distribution of the start points. The median thickness does not force the mask into a circular shape, but limits the influence of abnormally detected boundary points. Nevertheless, applying a common representative thickness may attenuate genuine direction-dependent variations in blooming thickness; therefore, anisotropic thickness estimation will be investigated in future work. Subsequently, the blooming start points and boundary points are connected between adjacent radial lines, and polygon filling is applied to generate a 2D blooming-distorted background mask (blooming mask). The region excluding the core region and the blooming-distorted background mask region is defined as the undistorted background mask. The blooming-distorted background mask (blooming mask) and undistorted background mask (background mask) generated in this manner are used in the subsequent depth correction module (depth-based) for depth correction.

### 2.6. Blooming-Region-Aware Directional Weighting Filter

**Figure 4:**
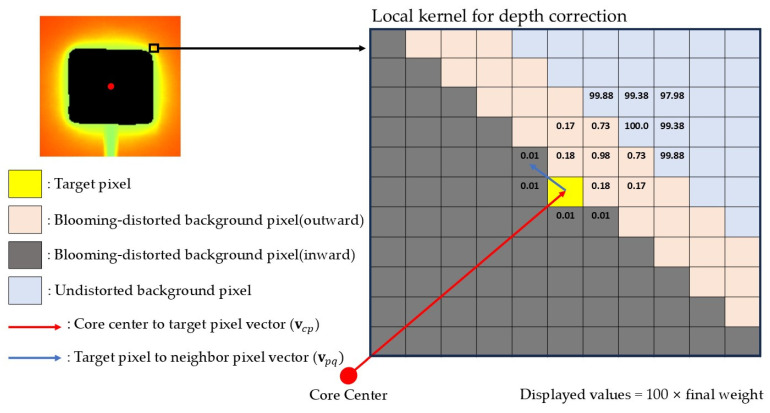
Core center-guided directional weighting for depth correction. For each target pixel, the final weight of each neighbor pixel in the local kernel is computed by combining the base weight, spatial distance weight, and core center-guided directional weight. The numerical values displayed in the kernel correspond to the final weights multiplied by 100 for visualization.

Module 4 in Figure 1, the blooming-region-aware directional weighting filter, performs multi-weight depth correction by applying a local kernel to distorted pixels inside the blooming mask on the depth map and using the pixel label-dependent base weight, directional weight, and Gaussian distance weight. The purpose of this module is to correct the depth inside the blooming-distorted background region so that it becomes closer to the surrounding undistorted background depth. Figure 4 shows the process in which the base weight, directional weight, and Gaussian distance weight are combined for the background neighbor pixel and the blooming neighbor pixel to compute the weights inside the local kernel.

First, each pixel inside the blooming-distorted background mask is set as a correction target, and a local window kernel is applied around that pixel. Neighbor pixels inside the local kernel are classified into background neighbor pixels and blooming neighbor pixels according to their mask labels. A background neighbor pixel is defined as a pixel inside the kernel that belongs to the undistorted background mask, and a high base weight is assigned to this pixel. In contrast, a blooming neighbor pixel is defined as a pixel inside the kernel that belongs to the blooming-distorted background mask, and a low base weight is assigned to this pixel. In this study, pixel label-dependent base weights of 1.0 and 0.01 are assigned to background neighbor pixels and blooming-distorted background pixels, respectively.

Even within the blooming-distorted background region, the degree of distortion may vary depending on the pixel location. In general, pixels closer to the core region tend to exhibit relatively severe depth distortion, whereas depth distortion tends to be relatively alleviated as the distance from the core region increases. Therefore, if neighbor pixels inside the local kernel are considered only based on spatial distance, inward neighbor pixels located in the core direction and outward neighbor pixels located in the background direction with respect to the correction target pixel may have similar influence on the correction result. To alleviate this issue, this study computes the directional relation between the vector from the core center to the target pixel and the vector from the target pixel to the neighbor pixel, and incorporates this relation as the directional weight. The unit vector from the core center to the target pixel and the unit vector from the target pixel to the neighbor pixel are defined as follows:

(1) $${\vec{v}}_{cp}=\frac{p-c}{\left\|p-c\right\|}$$

(2) $${\vec{v}}_{pq}=\frac{q-p}{\left\|q-p\right\|}$$

where the target pixel, which is the kernel center, is denoted as p, the neighbor pixel inside the local kernel is denoted as q, and the core center is denoted as c. Here, p−c represents the vector from the core center c to the target pixel p, and q−p represents the vector from the target pixel p to the neighbor pixel q.

Using the dot product of the two direction vectors, the directional weight is computed as follows:

(3) $$\phi \left(p,q\right)={\left[\max\left(0.01,{\vec{v}}_{cp}\cdot {\vec{v}}_{pq}\right)\right]}^{\gamma }$$

where γ is a parameter that controls the selectivity of the directional weight, and ϕ(p,q) denotes the directional weight. Since ${\vec{v}}_{cp}$ and ${\vec{v}}_{pq}$ are the unit vector from the core center to the target pixel and the unit vector from the target pixel to the neighbor pixel, respectively, the dot product of the two vectors is determined by the angle between ${\vec{v}}_{cp}$ and ${\vec{v}}_{pq}$. A larger dot product value indicates that the neighbor pixel q can be interpreted as an outward neighbor pixel and therefore has a higher directional weight. Conversely, a smaller dot product value indicates that the neighbor pixel can be interpreted as an inward neighbor pixel and therefore has a lower directional weight. In addition, the max(0.01, ⋅) operation serves as a lower bound to assign a small positive weight even when the directional weight becomes zero or negative. In this implementation, γ=5.0 was used.

The pixel label-dependent base weight and core center-guided directional weight are key components that distinguish the proposed blooming-region-aware directional weighting filter from a simple distance-based filter. If neighbor pixels inside the local kernel are considered only based on their spatial distance, it is difficult to account for the reliability difference between background neighbor pixels and blooming neighbor pixels. It is also difficult to account for the difference in distortion degree between core-side inward neighbor pixels and background-side outward neighbor pixels inside the blooming-distorted background region. In contrast, the proposed method assigns different base weights to background neighbor pixels and blooming neighbor pixels according to the mask labels obtained from the blooming region detection modules in Figure 1. In addition, to account for the geometric characteristic that depth distortion appears strongly around the core region inside the blooming-distorted background mask and is alleviated as the distance from the core region increases, the directional weight computed with respect to the core center is also applied. Through this process, among the blooming neighbor pixels inside the local kernel, the weights of core-side inward neighbors are limited, whereas background-side outward neighbors are used as relatively more reliable references. Therefore, the proposed method corrects the blooming-distorted background depth using the blooming-region-aware directional weighting filter, which considers not only the spatial distance of neighbor pixels but also their region labels and directionality with respect to the core center.

The pixel label-dependent base weight and directional weight defined above are components used to account for the mask label of each neighbor pixel and its directionality with respect to the core center. In addition, to account for the spatial distance between the target pixel and the neighbor pixel inside the local kernel, a Gaussian distance weight is applied to compute the final weight. The final weight is computed differently depending on whether the neighbor pixel is included in the blooming mask or the background mask, and it is expressed as follows:

(4) $$w\left(p,q\right)=\{\begin{array}{c} w_{g}\left(p,q\right),\;\;q\in \;G\;\\ w_{b}\left(p,q\right),\;\;q\in \;B \end{array}$$

where G and B denote the background mask and the blooming mask inside the local kernel, respectively. That is, q∈ G indicates that the neighbor pixel q belongs to the background mask, whereas q∈ B indicates that the neighbor pixel q belongs to the blooming mask. Equation (4) defines the application of different weight functions according to the mask label of each neighbor pixel. When the neighbor pixel q is an undistorted background neighbor pixel, $w_{g}\left(p,q\right)$ is used; when the neighbor pixel q is a blooming-distorted background neighbor pixel, $w_{b}\left(p,q\right)$ is used.

In Equation (4), $w_{g}\left(p,q\right)$ is the weight for the undistorted background neighbor pixel q, and it is computed as follows:

(5) $$w_{g}\left(p,q\right)=W_{IG}exp\left[\frac{-{\left(d_{g}\left(p,q\right)-d_{g}^{min}\right)}^{2}}{2\sigma_{s}^{2}}\right]$$

where $d_{g}\left(p,q\right)$ denotes the image-plane distance between the correction target pixel p and the undistorted background neighbor pixel q inside the local kernel, and $d_{g}^{min}$ denotes the image-plane distance from the target pixel p to the nearest undistorted background neighbor pixel inside the local kernel. $\sigma_{s}$ is a parameter that controls the decay of the Gaussian distance weight, and $W_{IG}$ is the pixel label-dependent base weight assigned to undistorted background neighbor pixels. In this implementation, the local kernel size was set to 11, $W_{IG}$ was set to 1.0, and $\sigma_{s}$ was set to 7.0. This setting is intended to strongly incorporate undistorted background neighbors, which are relatively less affected by blooming, as reliable references. In other words, background neighbors have high base reliability while having different weights according to their distance relationship with the target pixel inside the local kernel.

In Equation (4), $w_{b}\left(p,q\right)$ is the weight for the blooming-distorted background neighbor pixel q, and it is computed as follows:

(6) $$w_{b}\left(p,q\right)=W_{IB}exp\left(\frac{-d_{b}{\left(p,q\right)}^{2}}{2\sigma_{s}^{2}}\right)\phi \left(p,q\right)$$

where $d_{b}\left(p,q\right)$ denotes the image-plane distance between the correction target pixel p and the blooming-distorted background neighbor pixel q inside the local kernel, and $W_{IB}$ is the pixel label-dependent base weight assigned to blooming neighbors. The directional weight ϕ(p,q) in Equation (3) is also incorporated into the blooming weight. Through this formulation, the weights of core-side inward neighbor pixels, which have relatively severe depth distortion, are limited, whereas background-side outward neighbor pixels are used as relatively more reliable references. In this implementation, $W_{IB}$ was set to 0.01, and $\sigma_{s}$ was set to 7.0. This setting is intended to limit the reliability of blooming neighbor pixels, which are relatively strongly affected by blooming, while not completely excluding the depth information available inside the local kernel.

Using w(p,q) in Equation (4), the corrected depth of the correction target pixel p is computed using the weighted average of neighbor depths inside the local kernel, as follows:

(7) $$D_{corr}\left(p\right)=\frac{\sum_{q\in \Omega_{p}}w\left(p,q\right)D_{ref}\left(q\right)}{\sum_{q\in \Omega_{p}}w\left(p,q\right)}$$

where $D_{corr}\left(p\right)$ denotes the corrected depth value, $\Omega_{p}$ denotes the local kernel centered at the target pixel p, and $D_{ref}$ denotes the currently available depth value at the neighbor pixel q. That is, if q is a blooming pixel that has already been corrected, the corrected depth value is used; otherwise, the original depth value is used.

Depth restoration is not performed independently for all pixels inside the blooming mask at once. Instead, it is performed sequentially from blooming-distorted background pixels that are close to the background mask. To this end, the distance from each blooming-distorted background pixel to the nearest undistorted background pixel is computed, and pixels with smaller distances are corrected first. A pixel corrected earlier can then be used as a neighbor inside the local kernel when correcting a more inward blooming-distorted pixel. Accordingly, in Equation (7), $D_{ref}$ uses the corrected depth value when the neighbor pixel q is a blooming pixel that has already been corrected; otherwise, it uses the original depth value. Therefore, the proposed method can be interpreted as an inpainting process that progressively propagates corrected values into the blooming mask, starting from the surrounding undistorted background depth.

As a result, the proposed blooming-region-aware directional weighting filter is a multi-weight restoration process that jointly incorporates the pixel label-dependent base weight, core center-guided directional weight, and Gaussian distance weight by considering the structural characteristics of the blooming artifact. Specifically, different base weights are assigned to undistorted background neighbors and blooming-distorted neighbors, and the final weight is computed by jointly considering the directionality with respect to the core center and the distance between the target pixel and the neighbor pixel. Subsequently, a weighted average of the neighbor depths inside the local kernel is computed to correct the distorted depth inside the blooming-distorted background mask so that the corrected depth becomes closer to the surrounding undistorted background depth.

### 2.7. Pseudo Code

The overall procedure of the proposed method is summarized in Algorithm 1. Algorithm 1 presents the sequential workflow of the intensity-based blooming region detection module and the depth-based correction module. After performing radial line-based blooming mask reconstruction, the proposed method applies core center-guided directional weighting and multi-weight depth restoration to correct the distorted depth inside the blooming mask.
**Algorithm 1:** Proposed rule-based depth correction algorithm**Input**: Intensity map I and depth map D
**Output**: Corrected depth map $\hat{D}$ and blooming mask $M_{B}$ (1) Compute the saliency map S from I (2) Compute the Sobel-gradient magnitude map $G_{Sobel}$ from I (3) Identify the core mask $M_{C}$ from I (4) Estimate the core center c from $M_{C}$ using distance transform (5) **for** each radial direction $\theta_{k}$ do (6)  Generate the radial line $l_{k}$ from c (7)  Estimate the blooming start point $s_{k}$ on $l_{k}$ (8)  Estimate the boundary point $b_{k}$ on $l_{k}$ (9) **end for** (10) Compute the representative blooming thickness $t_{m}$ (11) Adjust each boundary point using $t_{m}$ (12) Reconstruct the blooming mask $M_{B}$ (13) Remove $M_{C}$ from $M_{B}$ (14) Define the background mask $M_{G}$ (15) Initialize the corrected depth map $\hat{D}$ with D (16) Sort pixels in $M_{B}$ from the background side to the core side (17) **for** each target pixel p in the sorted blooming pixels do (18) Define the local kernel $\Omega_{p}$ centered at p (19) Define the background neighbor set ($G_{p}\leftarrow \Omega_{p}\cap M_{G}$)  (20) Define the blooming neighbor set $\left(B_{p}\leftarrow \Omega_{p}\cap M_{B}\right)$ (21) **for** each neighbor pixel $q\in G_{p}$ do (22)  Compute $w_{g}\left(p,q\right)$ using base weight $W_{IG}$ and Gaussian distance weight (23)  Set w(p,q)← $w_{g}\left(p,q\right)$ (24) **end for** (25) **for** each neighbor pixel $q\in B_{p}$ do (26)  Compute ϕ(p,q) using core center-guided directional weighting (27)  Compute $w_{b}\left(p,q\right)$ using base weight $W_{IB}$, Gaussian distance weight, and ϕ(p,q) (28)  Set w(p,q) ← $w_{b}\left(p,q\right)$
 (29) **end for** (30) Update $\hat{D}\left(p\right)$ by weighted averaging neighboring depth values using w(p,q) (31) **end for** (32) **return** $\hat{D}$ and $M_{B}$


## 3. Performance Evaluation and Ablation Study

This section describes the experimental setup and evaluation metrics used for the performance evaluation and analyzes the repeatability of the GT and blooming depth measurements. For the comparison of depth correction methods, the same blooming mask generated by the blooming region detection module in Figure 1 was applied to all methods, such that only the depth correction method differed. In addition, the frame-to-frame repeatability of the depth measurements was evaluated using 30 consecutive GT and blooming depth frames acquired under the same static experimental conditions.

### 3.1. Experimental Setup, Evaluation Metrics, and Repeatability Analysis

**Figure 5:**
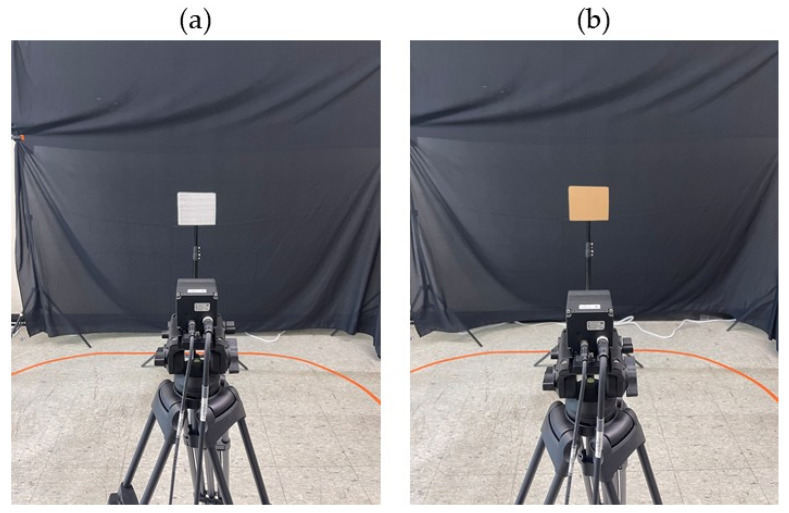
Experimental setup for paired acquisition of ground-truth and blooming-distorted depth maps. (**a**) Non-specular planar surface object used for ground-truth depth acquisition. (**b**) Same planar surface object with a high-reflectance target attached for blooming-distorted depth acquisition.

Figure 5 shows the experimental environment used for the performance evaluation and ablation study. A LUCID Helios2 ToF camera was used, and the GT depth map was acquired by capturing a non-specular planar surface object. Subsequently, while maintaining the same experimental conditions, including the camera position, object position, and background condition, a high-reflectance target was attached to the planar surface object to acquire a depth map in which the blooming artifact appeared. In this ablation study, based on the GT depth map, the blooming-distorted depth map before correction, referred to as the blooming depth map, and the corrected depth maps obtained by applying various depth correction methods were compared. In addition, for the qualitative comparison of the depth maps, the visualization scale was selected according to the depth range of each experimental condition, and the same scale was applied to the GT depth, blooming depth, and corrected depth within each comparison condition.

For the evaluation, the blooming depth map in which depth distortion occurred due to the high-reflectance target, the corrected depth map obtained by applying each method, and the GT depth map acquired under the corresponding non-blooming condition were used. Quantitative evaluation was performed within the blooming mask using pixels for which valid depth values were available in both the evaluated depth map and the corresponding GT depth map, excluding the core region. The MSE of each method is computed as follows:

(8) $$MSE=\frac{1}{\left|\Omega \right|}\sum_{p\in \Omega }{\left(D_{eval}\left(p\right)-D_{GT}\left(p\right)\right)}^{2}$$

where Ω denotes the evaluation region, and |Ω| denotes the number of pixels inside the evaluation region. $D_{eval}\left(p\right)$ denotes the depth map used for evaluation and includes both the blooming depth map before correction and the corrected depth maps obtained by applying each depth restoration method. $D_{GT}\left(p\right)$ denotes the GT depth map. The RMSE is computed as follows:

(9) $$RMSE=\sqrt{MSE}$$

In addition, the RMSE reduction was used to evaluate the error reduction rate of each correction method relative to the blooming depth before correction, as follows:

(10) $$\mathrm{RMSE}\;\mathrm{reduction}\left(\%\right)=\frac{\mathrm{RMS}E_{\mathrm{blooming}}-\mathrm{RMS}E_{\mathrm{corrected}}}{\mathrm{RMS}E_{\mathrm{blooming}}}\times 100$$

where $\mathrm{RMS}E_{\mathrm{blooming}}$ denotes the RMSE between the blooming depth map before correction and the GT depth map, and $\mathrm{RMS}E_{\mathrm{corrected}}$ denotes the RMSE between the corrected depth map obtained after applying each method and the GT depth map. A larger RMSE reduction value indicates that the corresponding method more effectively reduced the blooming-induced depth distortion. All MSE and RMSE values presented in the tables were rounded to three decimal places, whereas the RMSE reduction was calculated using the corresponding unrounded RMSE values and subsequently rounded to three decimal places. Therefore, even when the displayed MSE and RMSE values are identical, slight differences may appear in the RMSE reduction values.

**Figure 6:**
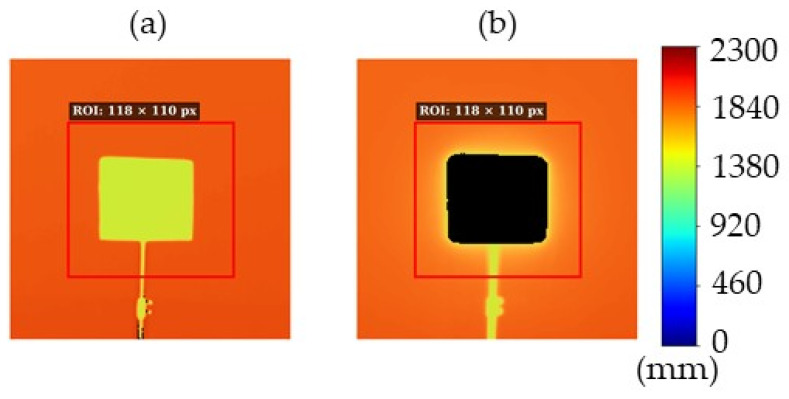
Fixed rectangular ROI used for the 30-frame repeatability analysis: (**a**) median GT depth and (**b**) median blooming depth. The same 118 × 110-pixel ROI covers the spatial extent corresponding to the core and blooming regions detected across the blooming depth frames.

To evaluate the frame-to-frame repeatability of the depth measurements, 30 consecutive GT and blooming depth frames were acquired under the same static experimental conditions. Figure 6 shows the pixel-wise median GT and blooming depth maps and the fixed ROI used for the repeatability analysis. The ROI was defined as a fixed rectangular region of 118 × 110 pixels that enclosed the union of the core and blooming regions detected across the 30 blooming depth frames. The same ROI was applied to all GT and blooming depth frames, and the mean depth within the ROI was calculated for each frame. Table 1 presents the overall mean and frame-to-frame sample standard deviation of the frame-wise ROI mean depths. For the GT depth frames, the mean depth was 1.683013 m, and the standard deviation was 0.002304 m, corresponding to approximately 0.137% of the mean depth. For the blooming depth frames, the corresponding values were 1.680509 m and 0.002642 m, respectively, with the standard deviation corresponding to approximately 0.157% of the mean depth. These results indicate that both the GT and blooming depth measurements exhibited high frame-to-frame repeatability within the evaluated ROI. The same 30 consecutive GT and blooming depth frames were also used for the frame-wise comparison of the depth correction methods presented in Section 3.4.

### 3.2. Ablation Study of Directional Weighting

**Figure 7:**
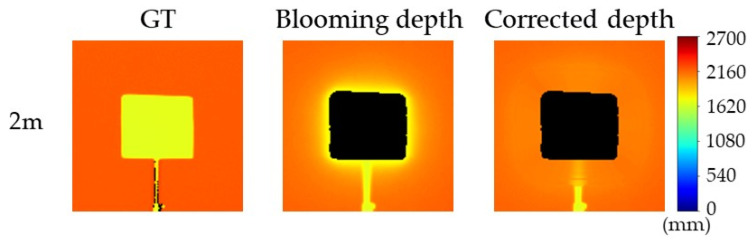
Depth maps under the 2.0 m black-background condition used for the directional-weight ablation and parameter sensitivity analyses.

This section evaluates the contribution of the directional weight to depth correction performance. Figure 7 shows the GT depth, blooming depth, and corrected depth obtained under the 2.0 m black-background condition, which was used for both the directional-weighting ablation in this section and the parameter sensitivity analysis in Section 3.3. To isolate the effect of the directional weight, the condition without directional weighting was compared with the full proposed method using the same input data, blooming mask, and evaluation mask. The pixel label-dependent base weight, Gaussian distance weight, and all other algorithm parameters were maintained identically under both conditions.

Table 2 compares the quantitative results obtained with and without directional weighting. Before correction, the MSE and RMSE of the blooming depth were 0.060 m^2^ and 0.245 m, respectively. Without directional weighting, the corrected MSE and RMSE were 0.051 m^2^ and 0.226 m, respectively, corresponding to an RMSE reduction of 7.787%. In contrast, the full proposed method reduced the MSE and RMSE to 0.011 m^2^ and 0.103 m, respectively, and achieved an RMSE reduction of 57.880%.

The full proposed method reduced the RMSE by approximately 54.3% compared with the condition without directional weighting. This result indicates that incorporating the radial characteristic of blooming, which spreads from the core center toward the surrounding background, plays a primary role in reducing the depth error. Specifically, the directional weight suppresses the contribution of distorted neighbor depths located toward the core and increases the relative contribution of neighbor depths located toward the background. The pixel label-dependent base weight and Gaussian distance weight were retained in both conditions to account for the lower reliability of blooming-distorted depth pixels and the local spatial continuity of the depth distribution, respectively. However, the limited RMSE reduction obtained without directional weighting indicates that their combination was insufficient to provide substantial correction without directional information under the evaluated condition.

### 3.3. Parameter Sensitivity Analysis

This section evaluates the sensitivity of the correction performance to variations in the major parameters of the proposed method: the saliency percentile, gradient threshold, directional exponent (γ), and Gaussian parameter (σ). All analyses used the 2.0 m black-background data shown in Figure 7 and the same fixed evaluation mask. A one-factor-at-a-time approach was employed, with reference values of 99.7, 300, 5, and 7 for the saliency percentile, gradient threshold, γ, and σ, respectively. The minimum consecutive low-gradient length, pixel label-dependent base weights, kernel size, and distance-transform criterion were empirically determined in preliminary experiments and held constant throughout the analysis. Because the same input data and evaluation mask were used, the MSE and RMSE before correction remained 0.060 m^2^ and 0.245 m, respectively, in all cases. The correction performance was evaluated against the GT depth using the MSE, RMSE, and RMSE reduction.

Table 3 presents the results obtained by varying the saliency percentile among 99.5, 99.7, and 99.9. All three values produced identical corrected MSE and RMSE values of 0.011 m^2^ and 0.103 m, respectively, and an RMSE reduction of 57.880%. Thus, the proposed method maintained stable correction performance over the evaluated range, and its midpoint, 99.7, was selected as the reference value.

Table 4 presents the results obtained at gradient thresholds of 100, 300, and 500. Thresholds of 100 and 300 produced identical corrected MSE and RMSE values of 0.011 m^2^ and 0.103 m, respectively, with an RMSE reduction of 57.880%. At 500, the corrected MSE and RMSE increased to 0.012 m^2^ and 0.109 m, respectively, and the RMSE reduction decreased to 55.593%. This decrease indicates that an excessively high threshold may exclude relatively weak intensity gradients during blooming-boundary detection. Because thresholds of 100 and 300 produced identical performance, 300 was selected to limit unnecessary responses to weak background gradients while avoiding the performance degradation observed at 500.

Table 5 presents the results obtained by varying γ among 1, 5, and 9. The directional exponent controls the influence of the directional agreement between a correction target pixel and its neighbor pixels. At γ=1, the corrected MSE and RMSE were 0.013 m^2^ and 0.112 m, respectively, with an RMSE reduction of 54.420%. Increasing γ to 5 reduced the MSE and RMSE to 0.011 m^2^ and 0.103 m, respectively, and increased the RMSE reduction to 57.880%. At γ=9, the rounded MSE and RMSE remained unchanged, and the RMSE reduction increased only marginally to 57.969%. Therefore, γ=5 was selected as the point at which the correction performance substantially stabilized without unnecessarily emphasizing the directional relationship.

Table 6 presents the results obtained by varying σ among 3, 7, and 11. The Gaussian parameter controls the spatial decay of the neighbor-pixel contribution with distance from the correction target pixel. At σ=3, the corrected MSE and RMSE were 0.011 m^2^ and 0.104 m, respectively, with an RMSE reduction of 57.629%. At σ=7 and 11, the corrected MSE and RMSE were both 0.011 m^2^ and 0.103 m, respectively, while the RMSE reductions were 57.880% and 57.926%. Although σ=11 produced the lowest unrounded RMSE, its difference from that obtained at σ=7 was only approximately 0.000111 m. Because increasing σ also reduces the local distance-dependent differences in the Gaussian weights, σ=7 was selected to preserve the local spatial weighting effect while providing substantially stabilized correction performance.

Overall, the sensitivity analysis showed that the proposed method maintained stable correction performance over the evaluated parameter ranges. The substantial difference between the results with and without directional weighting in Table 2 further indicates that directional weighting is the primary component contributing to RMSE reduction.

### 3.4. Comparison of Depth Correction Methods

This section compares five depth correction methods for correcting the distorted depth inside the blooming region. The compared methods consist of local plane fitting, nearest-background propagation, Laplacian/Poisson inpainting, iterative low-pass filtering, and the proposed blooming-region-aware directional weighting filter.

Local plane fitting is a method that applies a local kernel to each blooming-distorted background pixel, approximates the undistorted background pixels inside the kernel as a planar surface, and uses the depth value calculated from the corresponding plane as the corrected depth of the blooming-distorted background pixel [21,28]. Nearest-background propagation first identifies the nearest undistorted background pixel for each blooming-distorted pixel based on the distance transform [30]. The corresponding undistorted background depth value is then directly assigned as the corrected depth of the blooming-distorted pixel. Laplacian/Poisson inpainting is a method that sets the blooming-distorted region as an unknown region and reconstructs the depth distribution inside the blooming mask using the surrounding undistorted background depth as the boundary condition [33]. Iterative low-pass filtering progressively reconstructs the depth inside the blooming-distorted background region by repeatedly applying frequency-domain low-pass filtering with a progressively increasing cutoff frequency while preserving the valid measured depth outside the blooming mask [34]. The blooming-region-aware directional weighting filter is the method proposed in this study, which performs weighted averaging of neighbor depths inside the local kernel by jointly incorporating the pixel label-dependent base weight, directional weight, and Gaussian distance weight.

**Figure 8:**
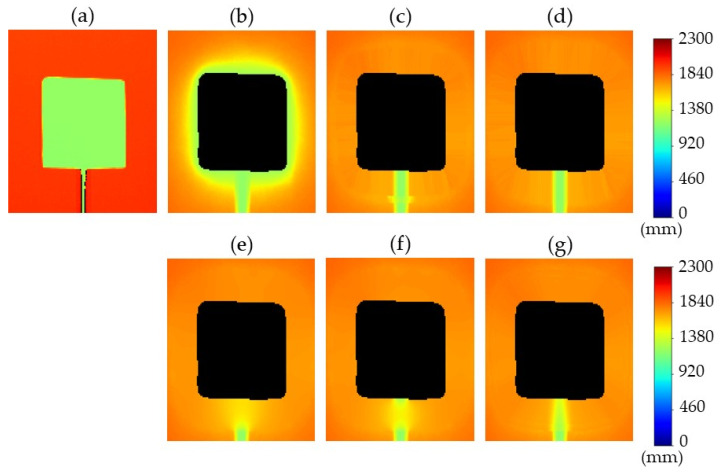
Qualitative comparison of depth restoration methods for the blooming-distorted region. (**a**) GT depth, (**b**) blooming-distorted depth, (**c**) local plane fitting, (**d**) nearest-background propagation, (**e**) Laplacian/Poisson inpainting, (**f**) iterative low-pass filtering [34], and (**g**) proposed blooming-region-aware directional weighting filter.

Figure 8 shows the GT depth, the blooming depth before correction, and the corrected depth maps obtained by applying the five depth correction methods. In the blooming depth before correction, unlike the GT depth map, the depth around the high-reflectance target was distorted due to the blooming artifact, and the results obtained by applying each depth correction method showed that the distortion caused by the surrounding blooming artifact was alleviated. However, the local plane fitting and nearest-background propagation methods retained some spatial texture inside the corrected region due to local fitting or nearest background assignment. The Laplacian/Poisson inpainting method tended to excessively smooth the corrected region. Iterative low-pass filtering also produced a smooth depth distribution inside the corrected region; however, an unnatural discontinuity appeared in the depth distribution of the supporting pole below the high-reflectance target. In contrast, the blooming-region-aware directional weighting filter generated a corrected depth map that was more naturally connected to the surrounding depth structure.

Table 7 compares the quantitative performance of the depth correction methods within the blooming-distorted region. Before correction, the blooming depth showed an MSE of 0.205 m^2^ and an RMSE of 0.453 m. Local plane fitting, nearest-background propagation, Laplacian/Poisson inpainting, and iterative low-pass filtering reduced the RMSE to 0.272 m, 0.269 m, 0.268 m, and 0.265 m, respectively, corresponding to RMSE reductions of 40.017%, 40.695%, 40.937%, and 41.530%. The proposed method achieved the lowest MSE and RMSE of 0.064 m^2^ and 0.254 m, respectively, as well as the highest RMSE reduction of 44.025%, indicating that it restored the blooming-affected depth closer to the GT than the comparison methods.

To further evaluate the frame-to-frame consistency of the correction performance, the five depth correction methods were applied to the same 30 consecutive blooming depth frames used in the repeatability analysis in Section 3.1. The pixel-wise median of the 30 GT depth frames was used as the fixed GT reference. In addition, the union of the blooming masks detected across the 30 frames, excluding the union of the core regions and invalid GT pixels, was used as a fixed evaluation mask for all frames and methods. The RMSE reduction was calculated independently for each frame relative to the corresponding blooming depth before correction. Table 8 presents the mean and sample standard deviation of the RMSE reduction values obtained across the 30 frames.

The proposed method achieved the highest mean RMSE reduction of 47.396%, with a sample standard deviation of 1.565 percentage points. The mean RMSE reductions in the comparison methods ranged from 42.448% to 45.451%, indicating that the proposed method provided a 1.945–4.948 percentage-point greater mean reduction. The relatively small standard deviations observed across all methods indicate that the correction performance remained consistent over the 30 consecutive frames.

### 3.5. Discussion

The directional-weighting ablation showed that the directional weight was the primary contributor to the improvement in depth correction performance under the evaluated condition. Removing the directional weight while retaining the other two weights limited the RMSE reduction to 7.787%, whereas the full proposed method achieved 57.880% and reduced the RMSE by approximately 54.3% relative to the condition without directional weighting. These results confirm that incorporating the radial characteristic of blooming, which spreads from the core region toward the surrounding background, plays a key role in reducing the blooming-induced depth error. The retained pixel label-dependent base weight and Gaussian distance weight accounted for the lower reliability of blooming-distorted depth pixels and the local spatial continuity of the depth distribution, respectively, but their combination provided only limited improvement without directional information.

The parameter sensitivity analysis showed generally stable correction performance over the evaluated ranges. Variations in the saliency percentile did not change the correction results, and gradient thresholds of 100 and 300 produced identical performance, whereas a slight decrease was observed at 500. The performance improvements beyond γ=5 and σ=7 were also marginal. Accordingly, the reference values were selected by jointly considering the observed performance and the function of each parameter, with γ=5 and σ=7 representing the points at which the correction performance had substantially stabilized.

All comparison methods reduced the MSE and RMSE relative to the blooming-distorted depth, confirming that the surrounding undistorted background depth can be used as reference information for correction. However, local plane fitting and nearest-background propagation retained some local spatial texture, Laplacian/Poisson inpainting produced an excessively uniform depth distribution, and iterative low-pass filtering caused an unnatural discontinuity in the supporting-pole structure. In contrast, the proposed method generated a corrected depth map that was more naturally connected to the surrounding background and achieved the lowest MSE and RMSE of 0.064 m^2^ and 0.254 m, respectively, as well as the highest RMSE reduction of 44.025%.

The 30-frame evaluation supported the single-frame comparison: the proposed method achieved the highest mean RMSE reduction, while the sample standard deviations across all methods ranged from 1.334 to 1.565 percentage points, indicating limited frame-to-frame variability under the evaluated static condition. Taken together, the single-frame and 30-frame results indicate that the proposed method alleviated blooming-induced background depth distortion more effectively than the comparison methods by jointly incorporating the directional characteristic of blooming, neighbor-depth reliability, and spatial proximity.

## 4. Correction Performance Analysis Under Various Experimental Conditions

This section analyzes the correction performance of the proposed depth correction method according to variations in target distance, target direction, background SNR, target shape, exposure time, and scene complexity. Target distance and target direction change the intensity and distribution of the optical signal returning from the high-reflectance target to the camera, thereby affecting the blooming artifact and the resulting blooming-affected background depth distortion. In addition, changes in background SNR affect the stability of the surrounding background depth measurements, which in turn influences the correction performance. Therefore, this section analyzes the depth restoration performance according to the distance and direction of the high-reflectance target and also evaluates the correction performance according to background SNR under each condition. This section also analyzes the correction results under different target-shape and exposure-time conditions, and discusses the limitations identified in multiple-object, non-planar-background, and complex-background scenes. Quantitative evaluation was performed based on the MSE, RMSE, and RMSE reduction defined in Section 3, and the GT depth, blooming-distorted depth (blooming depth), and corrected depth are presented together to qualitatively analyze the changes in depth distortion before and after correction.

### 4.1. Distance-Dependent Correction Performance

**Figure 9:**
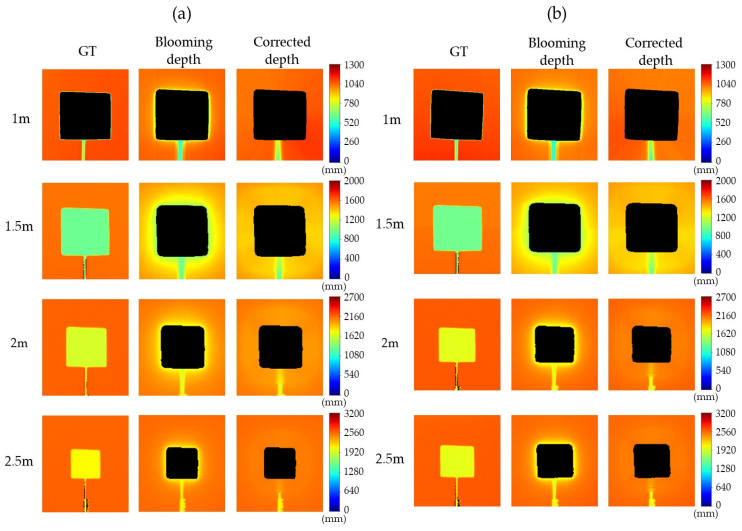
Qualitative comparison of depth correction results under different camera-to-background distances: (**a**) high-background-SNR condition (brown background) and (**b**) low-background-SNR condition (black background).

This section analyzes the depth correction performance of the blooming-region-aware directional weighting filter according to changes in target distance. The target distance conditions were set to 1.0 m, 1.5 m, 2.0 m, and 2.5 m, and the brown background, corresponding to the high-background-SNR condition, and the black background, corresponding to the low-background-SNR condition, were measured separately. Figure 9 shows the GT depth, blooming depth, and corrected depth according to changes in target distance under (a) the high-background-SNR condition and (b) the low-background-SNR condition. In this experiment, the distance refers to the distance between the camera and the background, and the high-reflectance target was positioned 0.5 m in front of the background toward the camera under all distance conditions.

Under all distance conditions, the background depth around the high-reflectance target was distorted by blooming compared with the GT depth. Under the 1.0 m condition, the target was located only 0.5 m from the camera, and invalid depth pixels with corresponding zero intensity values appeared even in parts of the low-reflectance target used for GT acquisition. This indicates that valid intensity and depth measurements were unavailable at these locations, presumably because of the measurement limitations of the ToF camera at extremely close range. The blooming-distorted background region was relatively extensive under the shorter-distance conditions, particularly at 1.5 m. As the distance increased to 2.0 and 2.5 m, the reflected optical signal weakened, and both the extent of the blooming-distorted region and the degree of depth distortion generally decreased. Consequently, the proposed method exhibited more stable correction performance at longer distances, where the blooming-induced depth distortion was relatively less severe.

Table 9 and Table 10 present the quantitative correction results according to changes in distance for the brown background, corresponding to the high-background-SNR condition, and the black background, corresponding to the low-background-SNR condition, respectively. Under both background conditions, the corrected depth showed lower MSE and RMSE than the blooming depth before correction, indicating that the blooming-region-aware directional weighting filter reduced the depth distortion caused by blooming under all distance conditions. Under the brown-background condition, the RMSE decreased from 0.080 m to 0.059 m, from 0.332 m to 0.223 m, from 0.246 m to 0.138 m, and from 0.217 m to 0.085 m at 1.0 m, 1.5 m, 2.0 m, and 2.5 m, respectively. The corresponding RMSE reductions were 26.199%, 32.738%, 43.938%, and 60.676%, respectively. Under the black-background condition, the RMSE decreased from 0.074 m to 0.057 m, from 0.270 m to 0.181 m, from 0.245 m to 0.103 m, and from 0.237 m to 0.092 m, respectively. The corresponding RMSE reductions were 23.380%, 32.922%, 57.880%, and 61.016%, respectively.

The quantitative results show that, under both background conditions, the RMSE reduction tended to increase as the distance increased, indicating that the relative correction effect of the proposed method became greater at longer distances. Under the 1.0 m condition, where the high-reflectance target was located at an extremely close distance of 0.5 m from the camera, the relative correction effect was limited under both background conditions. This can be attributed to the fact that, as the distance between the high-reflectance target and the camera decreases, the reflected optical signal received by the camera becomes stronger, and the depth distortion caused by blooming can occur more strongly. In contrast, as the distance increased, the distortion caused by blooming became relatively weaker, resulting in more stable correction performance. Differences according to the background SNR condition were also observed. Under the black-background condition, the RMSE before correction was higher at some distances than under the brown-background condition; however, after applying the proposed method, the corrected RMSE was reduced under all distance conditions. In particular, even under the black-background condition, the RMSE reduction ranged from 23.380% to 61.016%, indicating that the proposed method can alleviate blooming-induced background depth distortion even under low-background-SNR conditions.

### 4.2. Target Direction-Dependent Correction Performance

**Figure 10:**
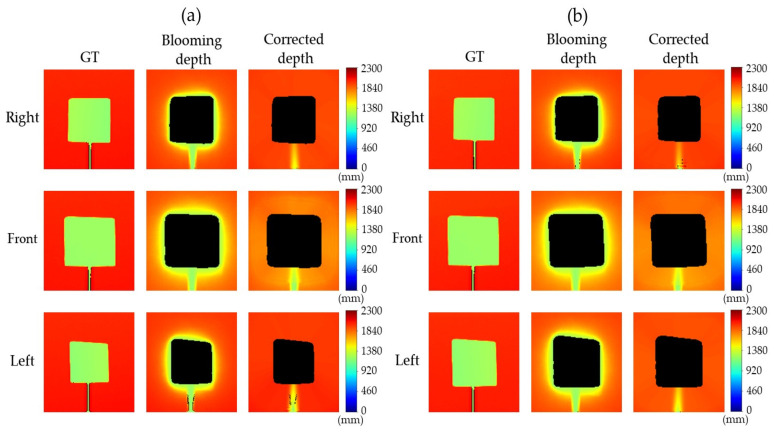
Qualitative comparison of depth correction results under different target directions: (**a**) high-background-SNR condition (brown background) and (**b**) low-background-SNR condition (black background).

This section analyzes the depth correction performance of the blooming-region-aware directional weighting filter according to changes in target direction. The target directions were set to right, front, and left. The front direction was defined as the 0° condition, in which the surface of the high-reflectance target directly faced the camera. The right and left directions were defined by rotating the high-reflectance target approximately 23° to the right and left, respectively, with respect to the optical axis of the camera. In addition, the brown background, corresponding to the high-background-SNR condition, and the black background, corresponding to the low-background-SNR condition, were measured separately. Figure 10 shows the GT depth, blooming depth, and corrected depth according to changes in target direction under (a) the high-background-SNR condition and (b) the low-background-SNR condition.

As shown in Figure 10, the background depth around the high-reflectance target was distorted by the blooming artifact under all target direction conditions. However, the distorted pattern differed depending on the target direction. In the front direction, because the target faced the camera directly, the blooming-distorted background region was formed relatively uniformly around the target. In contrast, in the right and left directions, the distorted region was biased or tilted toward one side depending on the target rotation direction. This can be attributed to the fact that the distribution of the reflected optical signal returning to the camera changes depending on the direction of the high-reflectance target, and consequently, the direction and extent to which the blooming artifact spreads into the surrounding background also change. In the corrected depth, the blooming-distorted background depth distortion around the target was generally alleviated under all direction conditions, and the depth distribution of the blooming-distorted background was restored to be more similar to the GT depth.

The quantitative results are presented in Table 11 and Table 12. Under the brown-background condition, the RMSE values between the blooming depth and the GT depth in the right, front, and left directions were measured as 0.289 m, 0.453 m, and 0.389 m, respectively, and decreased to 0.111 m, 0.254 m, and 0.092 m after applying the proposed method. The corresponding RMSE reductions were 61.560%, 44.025%, and 76.292%, respectively, with an average RMSE reduction of 60.626%. Under the black-background condition, the RMSE values in the right, front, and left directions were 0.260 m, 0.416 m, and 0.329 m before correction, respectively, and decreased to 0.075 m, 0.231 m, and 0.129 m after correction. The RMSE reductions were 71.389%, 44.487%, and 60.855%, respectively, with an average RMSE reduction of 58.910%.

The average RMSE reductions for the brown background and black background were 60.626% and 58.910%, respectively, showing similar values, and the average RMSE values after correction were also comparable at 0.152 m and 0.145 m, respectively. Under the tested conditions, the difference in background SNR appeared to have a limited effect on the overall correction performance. In contrast, differences among target directions were relatively distinct. Under both background conditions, the RMSE before correction was the largest in the front direction, which can be interpreted as being caused by stronger reflected optical signals received by the camera when the target faced the camera directly, resulting in stronger depth distortion due to blooming. In contrast, in the right and left directions, as the target was tilted with respect to the camera, the intensity of the reflected optical signal received by the camera was relatively lower than that in the front direction, and accordingly, the depth distortion caused by blooming tended to decrease. In fact, the RMSE values between the blooming depth before correction and the GT depth were the largest in the front direction, with 0.453 m for the brown background and 0.416 m for the black background, whereas they decreased to 0.289 m and 0.260 m in the right direction and to 0.389 m and 0.329 m in the left direction, respectively. These results indicate that the depth distortion caused by blooming was relatively weaker under side-view conditions than under the front-view condition, which consequently affected the correction performance. When the RMSE values after correction were compared, the front direction showed the highest values of 0.254 m and 0.231 m for the brown background and black background, respectively. In contrast, the RMSE values decreased to 0.111 m and 0.075 m in the right direction and to 0.092 m and 0.129 m in the left direction, respectively, indicating that the depth distortion caused by blooming was more effectively alleviated under side-view conditions. These residual errors indicate that, although the proposed method reduced the depth error in the front direction, its correction performance remained limited under the strong blooming-induced distortion caused by frontal reflection.

### 4.3. Correction Performance Under Target-Shape and Exposure-Time Variations

This section qualitatively analyzes the correction results of the proposed method under variations in high-reflectance target shape and camera exposure time. In each experiment, only the target shape or exposure time was varied, while the camera position, target position, and background condition were maintained.

**Figure 11:**
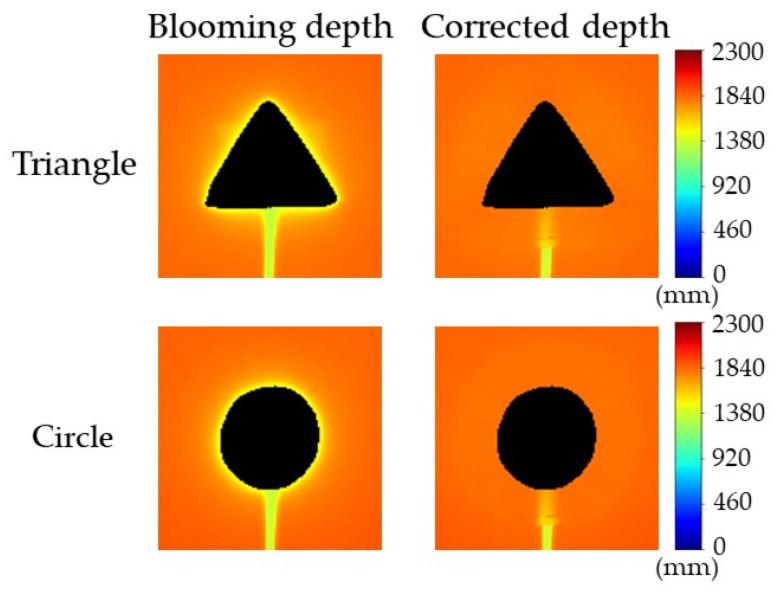
Qualitative comparison of blooming depth and corrected depth for triangular and circular high-reflectance targets.

Figure 11 shows the blooming depth and corrected depth obtained using triangular and circular high-reflectance targets. Similar to the rectangular target used in the preceding experiments, the blooming-induced depth distortion spread outward from the target boundary toward the surrounding background. However, its spatial distribution reflected the boundary geometry of each target: the distorted region was formed along the edges and vertices of the triangular target and along the continuous curved boundary of the circular target. After applying the proposed method, the extent of the distorted region was generally reduced for both target shapes. Because the proposed method estimates the blooming start and boundary points along radial lines generated with respect to the core center and incorporates the directional relationships among pixels, it does not assume a specific target shape. These results indicate that the proposed method can alleviate blooming-induced background depth distortion around targets with different boundary geometries.

**Figure 12:**
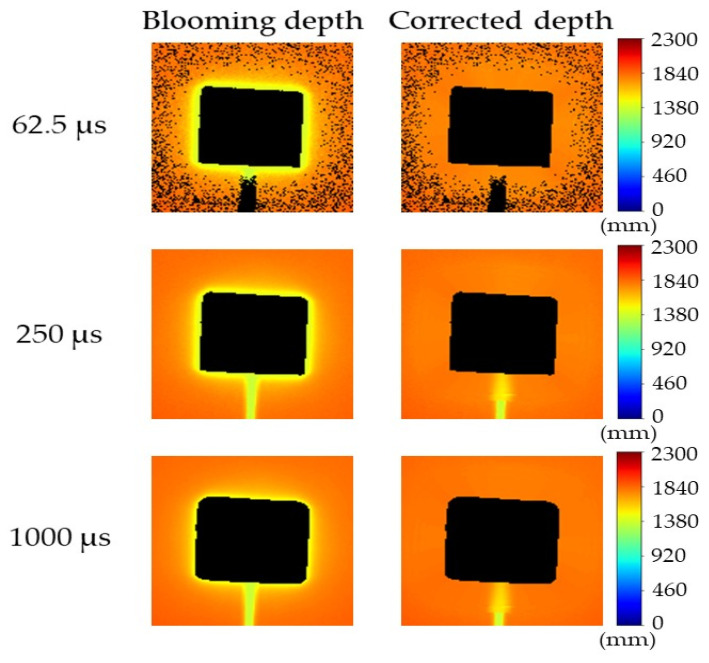
Qualitative comparison of blooming depth and corrected depth at exposure times of 62.5, 250, and 1000 μs.

Figure 12 shows the blooming depth and corrected depth acquired at exposure times of 62.5, 250, and 1000 μs, where 1000 μs corresponds to the exposure time used in Section 4.1 and Section 4.2. At 62.5 μs, numerous irregular or invalid depth pixels appeared throughout the background because the short exposure time reduced the received optical signal and background SNR. At 250 and 1000 μs, the background depth was measured more stably, although blooming-induced depth distortion remained around the high-reflectance target. In particular, the invalid core region centered on the high-reflectance target was wider at 1000 μs than at 62.5 and 250 μs. This was attributed to the increase in the received optical signal with exposure time, which expanded the region affected by saturation or invalid measurements around the high-reflectance target. After applying the proposed method, the blooming-induced depth distortion around the high-reflectance target was generally reduced under all three exposure-time conditions. However, some irregular or invalid background depth pixels caused by insufficient received signals at 62.5 μs, as well as the saturated or invalid core region, remained after correction. These regions are outside the correction scope of the proposed method, which is designed to correct the blooming-distorted background region containing valid depth measurements. Overall, the qualitative results indicate that the proposed method can alleviate blooming-induced background depth distortion for different target shapes and under different exposure-time conditions, although depth errors caused by insufficient received signals or invalid measurements remain outside its correction scope.

### 4.4. Limitations: Multiple-Object Case and Complex-Background Cases

The proposed method was designed to correct the blooming-distorted background region around a single high-reflectance target by applying a local kernel to each correction target pixel and calculating the corrected depth through weighted averaging of the neighbor depths. When other objects or non-planar background surfaces are located near the correction region, depth pixels belonging to different objects or surfaces may be included within the same kernel. Consequently, their different depth values may be jointly incorporated into the correction process, partially distorting the boundaries and depth structures of surrounding objects.

**Figure 13:**
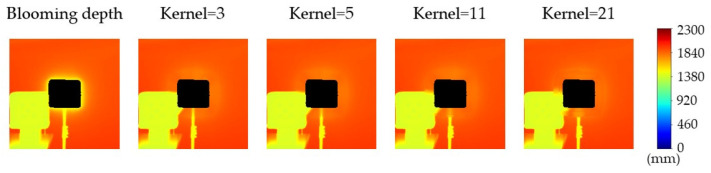
Effect of kernel size on depth correction in a multiple-object case.

Figure 13 shows the depth correction results according to kernel size in a multiple-object case. A small kernel limits the inclusion of depth pixels belonging to surrounding objects, whereas a larger kernel references a wider region and increases the possibility that adjacent-object depths are averaged with the background depth. Accordingly, the boundaries and depth structures of surrounding objects became progressively distorted as the kernel size increased. Therefore, the kernel size should be selected to secure sufficient background neighbor pixels while limiting the inclusion of adjacent-object depths.

**Figure 14:**
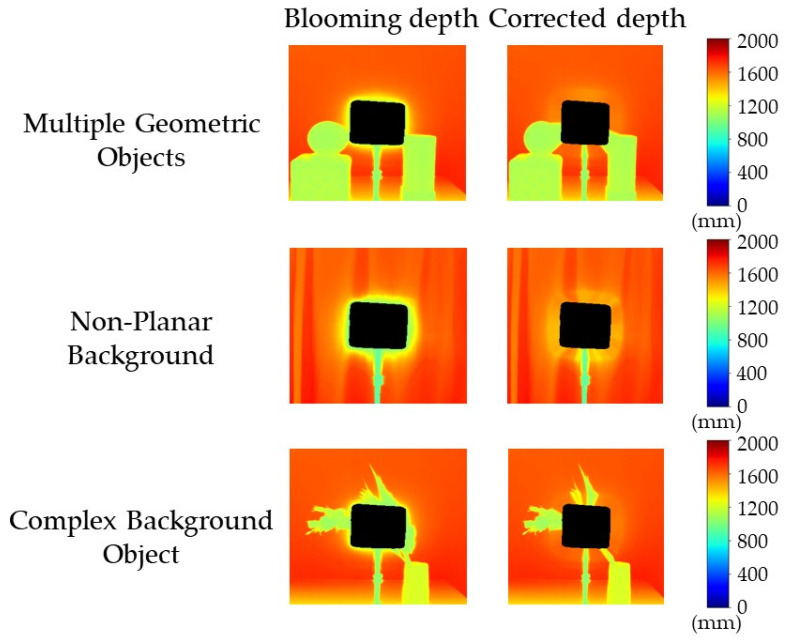
Qualitative depth correction results under multiple-object, non-planar-background, and complex-background conditions.

Figure 14 shows the blooming depth and corrected depth obtained under multiple-geometric-object, non-planar-background, and complex-background conditions. Based on the results in Figure 13, the kernel size was set to 3 for all conditions in Figure 14 to reduce the inclusion of depth pixels belonging to adjacent objects or different background surfaces. The proposed method generally reduced the blooming-induced depth distortion around the high-reflectance target. However, some adjacent-object boundaries and depth distributions were affected under the multiple-geometric-object condition. Accurate reconstruction was also more difficult under the non-planar-background condition because surfaces at different depths could be jointly incorporated into the correction process. Under the complex-background condition, some corrected regions did not transition smoothly to the surrounding background depth because of the complex object structures.

Complex scenes may also contain intensity variations and gradients unrelated to blooming because of object boundaries and surface structures. If these gradients are detected as blooming boundaries, the blooming mask may extend beyond the actual distorted region and include regions that do not require correction. When the reference gradient threshold of 300 was applied to the scenes in Figure 14, excessive expansion of the blooming mask was observed, particularly under the complex-background condition. Therefore, the gradient threshold was increased to 1500 for all conditions in Figure 14 to limit excessive blooming-region detection.

These results indicate that the proposed method can reduce blooming-induced depth distortion under the evaluated complex-scene conditions, although its performance may be limited by adjacent objects, non-planar surfaces, and complex intensity structures. The kernel size of 3 and gradient threshold of 1500 used in Figure 14 were selected for the evaluated scenes and may not be optimal for other scene configurations. Therefore, future work should adaptively determine these parameters based on surrounding-object boundaries, background geometry, and intensity distribution.

## 5. Conclusions and Future Work

In this paper, a blooming-region-aware directional weighting filter was proposed to correct blooming-induced background depth distortion occurring around a high-reflectance target. Deep learning-based depth correction methods have the advantage of learning complex artifact patterns when a sufficient training dataset is available [12,13,15,35,36,37,38]. However, it is difficult to acquire a large-scale dataset consisting of depth maps containing blooming artifacts and the corresponding GT depth maps, and diverse training data must also be constructed according to the target distance, target direction, background SNR, target shape, and exposure-time condition [29,36,38]. In addition, previous studies have mainly focused on flying pixel correction, depth denoising, reflective noise removal, blooming artifact pixel estimation, or camera-internal scattering compensation, whereas studies that directly correct localized blooming-distorted background depth around high-reflectance targets remain limited [10,12,13,14,15,16,17,19,20,21,29].

Considering these limitations, this study proposed a blooming-region-aware directional weighting filter that corrects blooming-distorted background depth using only the intensity map and depth map acquired from a single AMCW-ToF camera. The proposed method consists of intensity-based blooming detection and multi-weight depth restoration. It detects the blooming-distorted background region around a high-reflectance target and computes the corrected depth based on the surrounding undistorted background depth. The directional-weighting ablation showed that removing the directional weight resulted in an RMSE reduction of only 7.787%, whereas the full proposed method achieved 57.880%, confirming the primary contribution of the directional weight. The parameter sensitivity analysis showed generally stable correction performance within the evaluated ranges. In comparison with other depth correction methods, the proposed method achieved the lowest MSE and RMSE of 0.064 m^2^ and 0.254 m, respectively, and the highest RMSE reduction of 44.025%.

Across the distinct quantitative conditions involving different target distances, target directions, and background SNRs, the proposed method reduced both the MSE and RMSE, achieving an average RMSE reduction of 49.811% and a maximum of 76.292%. The qualitative results further showed reduced blooming-induced depth distortion for triangular and circular targets and at exposure times of 62.5, 250, and 1000 μs. However, saturated or invalid core regions and irregular or invalid background depth pixels caused by insufficient received signals remain outside the correction scope of the proposed method.

Because the proposed method is rule-based, its performance may depend on parameters such as the saliency percentile, gradient threshold, kernel size, and weighting parameters. In complex backgrounds, unrelated intensity gradients may cause the blooming mask to extend beyond the actual distorted region, whereas in multiple-object scenes, adjacent-object depths may be incorporated into the local kernel and distort surrounding depth structures. Future work should therefore develop adaptive methods for determining the gradient threshold and kernel size, together with a hyperparameter optimization framework that adjusts the major parameters according to the scene conditions.

This study primarily evaluated scenes containing a single high-reflectance target. Further validation is therefore required in multi-reflector or multi-blooming-target scenes containing multiple high-reflectance targets. In such scenes, the proposed method could be extended by separating the blooming candidate region around each high-reflectance target using object-detection-based bounding boxes and independently applying the proposed intensity-based blooming detection and multi-weight depth restoration to each region [39].

## Figures and Tables

**Table 1:** Repeatability of GT and blooming depth measurements over 30 consecutive frames within the fixed rectangular ROI.

Depth Map	Number of Frames	ROI Size (Pixels)	Mean Depth (m)	Frame-to-Frame Sample Standard Deviation (m)
GT depth	30	118 × 110	1.683013	0.002304
Blooming depth	30	118 × 110	1.680509	0.002642

**Table 2:** Quantitative comparison of depth correction performance with and without directional weighting.

	Blooming Depth-GT		Corrected Depth-GT		
Ablation Condition	MSE ($m^{2}$)	RMSE (m)	MSE ($m^{2}$)	RMSE (m)	RMSE Reduction (%)
Without directional weighting	0.060	0.245	0.051	0.226	7.787
Proposed method	0.060	0.245	0.011	0.103	57.880

**Table 3:** Effect of saliency percentile on depth correction performance.

	Blooming Depth-GT		Corrected Depth-GT		
Saliency Percentile	MSE ($m^{2}$)	RMSE (m)	MSE ($m^{2}$)	RMSE (m)	RMSE Reduction (%)
99.5	0.060	0.245	0.011	0.103	57.880
99.7	0.060	0.245	0.011	0.103	57.880
99.9	0.060	0.245	0.011	0.103	57.880

**Table 4:** Effect of gradient threshold on depth correction performance.

	Blooming Depth-GT		Corrected Depth-GT		
Gradient Threshold	MSE ($m^{2}$)	RMSE (m)	MSE ($m^{2}$)	RMSE (m)	RMSE Reduction (%)
100	0.060	0.245	0.011	0.103	57.880
300	0.060	0.245	0.011	0.103	57.880
500	0.060	0.245	0.012	0.109	55.593

**Table 5:** Effect of directional exponent on depth correction performance.

	Blooming Depth-GT		Corrected Depth-GT		
Directional Exponent (γ)	MSE ($m^{2}$)	RMSE (m)	MSE ($m^{2}$)	RMSE (m)	RMSE Reduction (%)
1	0.060	0.245	0.013	0.112	54.420
5	0.060	0.245	0.011	0.103	57.880
9	0.060	0.245	0.011	0.103	57.969

**Table 6:** Effect of Gaussian parameter on depth correction performance.

	Blooming Depth-GT		Corrected Depth-GT		
Gaussian Parameter (σ)	MSE ($m^{2}$)	RMSE (m)	MSE ($m^{2}$)	RMSE (m)	RMSE Reduction (%)
3	0.060	0.245	0.011	0.104	57.629
7	0.060	0.245	0.011	0.103	57.880
11	0.060	0.245	0.011	0.103	57.926

**Table 7:** Quantitative comparison of depth restoration methods for the blooming-distorted region.

Method	MSE ($m^{2}$)	RMSE (m)	RMSE Reduction (%)
Blooming-distorted depth	0.205	0.453	-
Local plane fitting	0.074	0.272	40.017
Nearest-background propagation	0.072	0.269	40.695
Laplacian/Poisson inpainting	0.072	0.268	40.937
Iterative low-pass filtering [34]	0.070	0.265	41.530
Proposed method	0.064	0.254	44.025

**Table 8:** Mean and sample standard deviation of the RMSE reduction achieved by each depth correction method over 30 consecutive frames acquired under the same static experimental condition.

Method	Mean RMSE Reduction (%)	Sample Standard Deviation (Percentage Points)
Local plane fitting	42.448	1.374
Nearest-background propagation	42.623	1.334
Laplacian/Poisson inpainting	45.237	1.536
Iterative low-pass filtering [34]	45.451	1.542
Proposed method	47.396	1.565

**Table 9:** Quantitative comparison of depth correction performance under different target distances in the high-background-SNR condition (brown background).

	Blooming Depth-GT		Corrected Depth-GT		
Distance	MSE ($m^{2}$)	RMSE (m)	MSE ($m^{2}$)	RMSE (m)	RMSE Reduction (%)
1 m	0.006	0.080	0.003	0.059	26.199
1.5 m	0.110	0.332	0.050	0.223	32.738
2 m	0.061	0.246	0.019	0.138	43.938
2.5 m	0.047	0.217	0.007	0.085	60.676

**Table 10:** Quantitative comparison of depth correction performance under different target distances in the low-background-SNR condition (black background).

	Blooming Depth-GT		Corrected Depth-GT		
Distance	MSE ($m^{2}$)	RMSE (m)	MSE ($m^{2}$)	RMSE (m)	RMSE Reduction (%)
1 m	0.006	0.074	0.003	0.057	23.380
1.5 m	0.073	0.270	0.033	0.181	32.922
2 m	0.060	0.245	0.011	0.103	57.880
2.5 m	0.056	0.237	0.009	0.092	61.016

**Table 11:** Quantitative comparison of depth correction performance under different target directions in the high-background-SNR condition (brown background).

	Blooming Depth-GT		Corrected Depth-GT		
Direction	MSE ($m^{2}$)	RMSE (m)	MSE ($m^{2}$)	RMSE (m)	RMSE Reduction (%)
Right	0.084	0.289	0.012	0.111	61.560
Front	0.205	0.453	0.064	0.254	44.025
Left	0.151	0.389	0.009	0.092	76.292

**Table 12:** Quantitative comparison of depth correction performance under different target directions in the low-background-SNR condition (black background).

	Blooming Depth-GT		Corrected Depth-GT		
Direction	MSE ($m^{2}$)	RMSE (m)	MSE ($m^{2}$)	RMSE (m)	RMSE Reduction (%)
Right	0.068	0.260	0.006	0.075	71.389
Front	0.173	0.416	0.053	0.231	44.487
Left	0.108	0.329	0.017	0.129	60.855

## Data Availability

The data supporting the findings of this study are available upon reasonable request.
